# Evolution and cancer: a mathematical biology approach

**DOI:** 10.1186/1745-6150-5-29

**Published:** 2010-04-20

**Authors:** Marek Kimmel

**Affiliations:** 1 Department of Statistics, Rice University, Houston, TX 77005, USA; 2 Systems Engineering Group, Silesian University of Technology, 44-100 Gliwice, Poland

## Evolution and cancer: a mathematical biology approach

This thematic series is concerned with various ways Evolution is intertwined with cancer. A group of cancer researchers and mathematical modelers, with an interest in the subject, was invited to contribute papers on topics ranging from carcinogenesis through progression of cancer through therapy. The resulting collection of ten papers is briefly introduced below. Publication of the series was planned to coincide with Darwin's Year, 2009, however various obstacles delayed publication.

Mathematical modeling of processes related to cancer evolution has come of age. What used to be a mathematical metaphor or speculation, has become progressively more infused with genetic and biological details and is reconcilable with epidemiology of a given cancer. The seminal biological discoveries, which lead to paradigm changes in modeling, include the concept of cancerization field, which puts into question the clonal carcinogenesis. Another set of discoveries includes genes with key roles in the regulation of DNA repair and genome expression that are often mutated in cancers, such as BRCA1, BRCA2 or P53, and understanding signaling pathways disrupted by mutations in these genes. Still another category of findings concerns tumor metabolism, which includes cell functioning under anoxic conditions. Finally, the understanding of structural issues such as vascularization of solid tumors, mechanisms of metastasis and invasion and emergence of resistance brought a totally different perspective on modeling of cancer growth and progression. In all these processes, well-known evolutionary forces such as mutation and selection play major roles. They are modulated and channeled both by the natural environment in which tumors exist, but also by medical intervention.

A review of current work on modeling of the role evolution plays in cancer is the subject of the Thematic Series. We briefly review the papers, assigning them idiosyncratically to 3 different categories.

### Carcinogenesis, evolution and emergence of cancer

The opening paper by Little [1] is a critical review of biology of cancer and argues based on evidence adduced that its development can be modeled as a somatic cellular Darwinian evolutionary process. A variety of quasi-mechanistic models of carcinogenesis are reviewed, all based on this somatic Darwinian evolutionary hypothesis; in particular, the multi-stage model of Armitage and Doll, the two-mutation model of Moolgavkar, Venzon, and Knudson (MVK), the generalized MVK model of Little and various further generalizations of these incorporating effects of genomic instability.

Following these basically clonal models, the paper by Agur et al. [2] discusses tumorigenesis as triggered by disruption of a quorum sensing mechanism, using a simple discrete model corroborated by experiments in mammary cancer stem cells. Application of this theory to a cellular automata model of stem cell development in disrupted environments, shows a sharply dichotomous growth dynamics: maturation within 50-400 cell-cycles, or immortalization. This dichotomy is mainly driven by intercellular communication, low values of which cause perpetual proliferation.

The subsequent contribution by Thalhauser et al. [3] shifts emphasis to selection in spatial stochastic models of cancer. The thesis is that migration is a key modulator of fitness. To study the selection dynamics in a heterogeneous spatial colony of cells, they use two spatial generalizations of the Moran process, which include cell divisions, death and migration. They find that repeated instances of large scale cell-death, such as might arise during therapeutic intervention or host response, strongly select for the migratory phenotype. The models help to explain how chemotherapy may provide a selection mechanism for highly invasive phenotypes.

### Darwinian evolution among cells and structures in cancer

This section contains works involved with evolutionary forces in later stages of cancer natural course. Smallbone et al. [4] study how episodic, transient systemic acidosis delays evolution of the malignant phenotype. The transition from premalignant to invasive tumour growth is a prolonged multistep process governed by phenotypic adaptation to changing microenvironmental selection pressures. Model simulations demonstrate that repeated episodes of transient systemic acidosis will interrupt critical evolutionary steps in the later stages of carcinogenesis resulting in substantial delay in the evolution to the invasive phenotype. The results suggest transient systemic acidosis may mediate the observed reduction in cancer risk associated with increased physical activity.

Enderling et al. [5] study tumor morphological evolution: directed migration and gain and loss of the self-metastatic phenotype. Considering only the properties of random migration in tumors composed of stem cells and committed cells, they recapitulate a characteristic clustering feature of invasive tumor growth, a property they attribute to "self-metastatic" growth. Furthermore, directed migration will result in loss of the invasive phenotype as the tumor approaches the attractor source.

Eikenberry et al. [6] consider the evolutionary impact of androgen levels on prostate cancer in a multi-scale mathematical model. Their results suggest that an aberrant androgen environment may delay progression to a malignant phenotype, but result in a more dangerous cancer should one arise. The model represents an initial framework for understanding the role of androgens in prostate cancer etiology, and it suggests that low androgen levels can increase selection for phenotypes resistant to hormonal therapy that may also be more aggressive. Moreover, clinical treatment with 5α-reductase inhibitors may increase the incidence of therapy resistant cancers.

Silva and Gatenby [7] present a theoretical quantitative model for evolution of cancer chemotherapy resistance. They propose that in order to understand the evolutionary dynamics that allow tumors to develop chemoresistance, a comprehensive quantitative model must be used to describe the interactions of cell resistance mechanisms and tumor microenvironment during chemotherapy. Results suggest that the maximum potential of a combined therapy may depend on how each of the drugs modifies the evolutionary landscape and that a rational use of these properties may prevent or at least delay relapse.

### Further implications for tumor dynamics and therapy

The last section includes models inspired by the evolutionary mechanisms discussed earlier on, and exploring their consequences for such specific processes as genome instability, oscillations in tumor growth and bone remodeling in multiple myeloma. Tan and Yan [8] introduce a new stochastic and state space model of human colon cancer incorporating multiple pathways. In this state space model, the stochastic system is represented by a stochastic model, involving 2 different pathways-the chromosomal instability pathway and the micro-satellite instability pathway; the observation, cancer incidence data, is represented by a statistical model. The model is applied to fit and analyze the SEER data of human colon cancers from NCI/NIH. The model not provides more insights into human colon cancer but also provides useful guidance for its prevention and control and for prediction of future cancer cases.

Stamper et al. [9] study evolution of oscillatory dynamics in a model of vascular tumor growth. By analysing a spatially uniform submodel, they identify regions of parameter space in which the combination of tumor cell proliferation and vessel occlusion give rise to sustained temporal oscillations in the tumor cell population and in the vessel density. Further, employing a combination of numerical and analytical techniques, they demonstrate how the spatio-temporal dynamics of the untreated tumor may influence its response to chemotherapy.

Ayati et al. [10] analyze a mathematical model of bone remodeling dynamics for normal bone cell populations and myeloma bone disease. Multiple myeloma is a hematologic malignancy associated with the development of a destructive osteolytic bone disease. The model examines the critical signaling between osteoclasts (bone resorption) and osteoblasts (bone formation). The therapeutic effects of targeting both myeloma cells and cells of the bone marrow microenvironment on these dynamics are examined.

Cancer is, in some sense, a condensed-time laboratory of evolution. Dedifferentiated cells form colonies that survive in hostile environment, and they evolve new metabolic circuits and aggression and resistance mechanisms. They also can muster cooperation of fibroblasts and lymphocytes and attract blood vessels. Modeling of these phenomena, well underway, will also help understand basic mechanisms of life. Taken together, the papers in this thematic series illustrate the substantial progress that occurred over the period of past decade. Qualitative and quantitative understanding of cancer is a necessary condition for engineering approaches to fight it. These latter are still scarce.
